# Anti‐inflammaging effects of human alpha‐1 antitrypsin

**DOI:** 10.1111/acel.12694

**Published:** 2017-10-17

**Authors:** Ye Yuan, Benedetto DiCiaccio, Ying Li, Ahmed S. Elshikha, Denis Titov, Brian Brenner, Lee Seifer, Hope Pan, Nurdina Karic, Mohammad A. Akbar, Yuanqing Lu, Sihong Song, Lei Zhou

**Affiliations:** ^1^ Department of Pharmaceutics University of Florida Gainesville FL USA; ^2^ Department of Molecular Genetics & Microbiology University of Florida Gainesville FL USA; ^3^ University of Florida Genetics Institute Gainesville FL USA; ^4^ UF Health Cancer Center Gainesville FL USA

**Keywords:** *Drosophila*, human alpha‐1 antitrypsin, inflammaging, NF‐κB, innate immune response, senescence‐associated secretory phenotype

## Abstract

Inflammaging plays an important role in most age‐related diseases. However, the mechanism of inflammaging is largely unknown, and therapeutic control of inflammaging is challenging. Human alpha‐1 antitrypsin (hAAT) has immune‐regulatory, anti‐inflammatory, and cytoprotective properties as demonstrated in several disease models including type 1 diabetes, arthritis, lupus, osteoporosis, and stroke. To test the potential anti‐inflammaging effect of hAAT, we generated transgenic *Drosophila* lines expressing hAAT. Surprisingly, the lifespan of hAAT‐expressing lines was significantly longer than that of genetically matched controls. To understand the mechanism underlying the anti‐aging effect of hAAT, we monitored the expression of aging‐associated genes and found that aging‐induced expressions of *Relish* (NF‐ĸB orthologue) and *Diptericin* were significantly lower in hAAT lines than in control lines. RNA‐seq analysis revealed that innate immunity genes regulated by NF‐kB were significantly and specifically inhibited in hAAT transgenic *Drosophila* lines. To confirm this anti‐inflammaging effect in human cells, we treated X‐ray‐induced senescence cells with hAAT and showed that hAAT treatment significantly decreased the expression and maturation of IL‐6 and IL‐8, two major factors of senescence‐associated secretory phenotype. Consistent with results from *Drosophila,* RNA‐seq analysis also showed that hAAT treatment significantly inhibited inflammation related genes and pathways. Together, our results demonstrated that hAAT significantly inhibited inflammaging in both *Drosophila* and human cell models. As hAAT is a FDA‐approved drug with a confirmed safety profile, this novel therapeutic potential may make hAAT a promising candidate to combat aging and aging‐related diseases.

## Introduction

Inflammation is required for organisms to fight with endogenous and exogenous pathogens. However, chronic inflammation sustained longer than necessary causes tissue damage and microenvironment impairment (Franceschi, 2007). One hallmark of aging is low‐grade, persistent, and ‘sterile’ inflammation, which is termed ‘inflammaging’ (Franceschi, 2007). Inflammaging has been implicated in most if not all of the aging‐related diseases, such as type II diabetes and cardiovascular diseases (Franceschi & Campisi, 2014). Although inflammation is an obvious target for preventing aging‐related diseases, it is challenging to develop a therapeutic approach to achieve this goal.

Several mechanisms were postulated as the cause of inflammaging (Franceschi & Campisi, 2014). Cellular debris accumulated with aging may function as endogenous stimulants of innate immunity. Alternatively, inflammatory factors secreted by senescent cells may be responsible for the ‘sterile’ inflammation. Among all the causes, cell senescence plays an important role and has been linked to multiple aging‐associated diseases (He & Sharpless, 2017). Depletion of senescent cells by targeting p16INK4A‐expressing cells in C57BL/6 mice increased the median lifespan, delayed cataract formation and decreased sclerotic glomeruli formation (Baker *et al*., 2016). A characteristic of senescence is the elevated expression of inflammatory cytokines and chemokines, growth factors, and proteases, which is termed senescence‐associated secretory phenotype (SASP) (He & Sharpless, 2017). The chronic inflammation induced by SASP is associated with aging‐related degenerative diseases, fibrotic pulmonary disease, and cancer (Coppe *et al*., 2008; Franceschi & Campisi, 2014; Schafer *et al*., 2017). DNA damage response and its downstream GATA4, p38MAPK, and mTOR pathways regulate SASP (Rodier *et al*., 2009; Freund *et al*., 2011; Kang *et al*., 2015; Laberge *et al*., 2015). All of these pathways converge on NF‐ĸB, the master transcription regulator that controls the expression of immune‐responsive genes (Zhang *et al*., 2013; Guo *et al*., 2014). A recent study showed SASP gene expression required cGMP‐AMP (cGAMP) synthase (cGAS) in mouse and human senescence model *in vitro* induced by DNA damage and ionizing radiation (Yang *et al*., 2017). Knock down H2A.J, a variant of histone H2A, down‐regulated SASP gene expression and protein secretion in senescent fibroblast (Contrepois *et al*., 2017), suggesting DNA chromatin may also participate in SASP regulation.

Human AAT (hAAT), a 52 kD glycoprotein in circulation, is primarily synthesized in the liver. IL‐6 or lipopolysaccharide (LPS) can stimulate the expression of hAAT (Perlmutter & Punsal, 1988). Human AAT has serine proteinase inhibitor activity, which can inhibit neutrophil elastase, proteinase 3, and cathepsin G. Human AAT can be chemically modified by nitric oxide (NO) and exhibits antibacterial and cysteine protease inhibitor activities (Miyamoto *et al*., 2000). We and others have shown that hAAT directly inhibits caspase‐3 and prevents apoptosis (Petrache *et al*., 2006; Zhang *et al*., 2007). Increasing evidence indicates that hAAT is a multifunctional protein and may play an important role in modulating the immune system. First, application of hAAT has been shown to alleviate symptoms in many disease models whereby immunity and inflammation were implicated, such as type 1 diabetes (Lu *et al*., 2006; Zhang *et al*., 2007; Ma *et al*., 2010), islet cell transplantation (Lewis *et al*., 2008), rheumatoid arthritis (Grimstein *et al*., 2010, 2011), stroke (Moldthan *et al*., 2014), and bone loss (Cao *et al*., 2011). A recent study indicated AAT treatment inhibited instant blood‐mediated inflammatory reaction (IBMIR) and islet apoptosis after islet cell transplantation (Wang *et al*., 2017). In addition, human AAT suppresses TNF and MMP‐12 production and enhances IL‐10 secretion in macrophages by increasing cAMP levels and activating cAMP‐dependent protein kinase A (Churg *et al*., 2007; Janciauskiene *et al*., 2007; Subramaniyam *et al*., 2008). While these evidences strongly point to the role of hAAT in modulating immunity and inflammation, it has never been shown to affect aging‐related inflammation.

In this study, we resorted to taking advantage of the genetic accessibility of *Drosophila* to explore the potential anti‐inflammaging effect of hAAT. Major regulatory pathways in innate immunity are highly conserved between *Drosophila* and vertebrates (Buchon *et al*., 2014). As a result, it is widely used as a model in aging‐related immune regulation studies (Guo *et al*., 2014) and has led to many seminal findings in this field. The Gal4‐UAS system with tissue‐specific promoter can provide robust spatial and temporal control of transgene expression. However, genetic variation due to strain background and recombination may complicate the interpretation. The Geneswitch system serves as a nice complement using mifepristone‐activated Gal4 (Nicholson *et al*., 2008), which has been extensively used in aging‐associated studies (Blice‐Baum *et al*., 2017).

## Results

### Systemic expression of hAAT extended the lifespan of *Drosophila*

**Figure 1:**
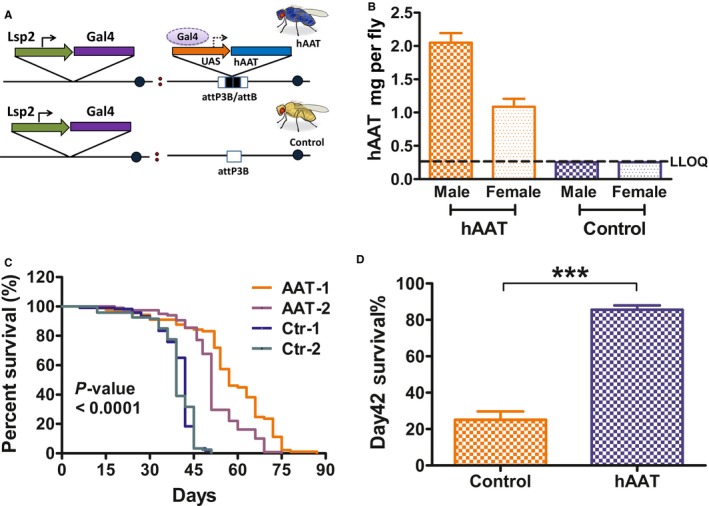
Transgenic expression of hAAT extended the lifespan of Gal4‐UAS‐hAAT transgenic *Drosophila*. (A) hAAT transgenic flies contain UAS‐hAAT and Lsp2‐Gal4 genes. Lsp2 drives Gal4 expression in the fat body (analogous to liver). Gal4 binds to UAS and activates the transcription of the hAAT gene. The control lines were generated in parallel and contain Lsp‐Gal4 and the docking site. Two independent lines of each genotype were generated. All experiments were processed with the independent lines and data was grouped and analyzed. (B) hAAT in transgenic flies was detected by ELISA. Each bar represents the average data from 2 to 3 detections. (C) Survival curve of Gal4‐UAS‐hAAT transgenic male flies (*n* = 89–120). (D) The percentage of files survived at day 42 after eclosion (8–10/vial, 16–20 vials per group). Percentage of survival was analyzed by both log‐rank (Mantel–Cox) test and log‐rank test for trend. Day 42 survival was analyzed by two‐tailed unpaired *t*‐test. ***P ˂ 0.001.

In humans, AAT is mainly synthesized and released from the liver. To explore the impact of systemic hAAT expression in *Drosophila*, we used the Gal4‐UAS system to direct the expression of hAAT from the fat body, which is analogous to the liver in mammals. We created independent recombination strains of the genotype lsp2‐Gal4:UAS‐hAAT as hAAT‐expressing lines. To control for genetic background, we generated genetic recombination with the lsp2‐Gal4 line and the docking strain that was used to generate the UAS‐hAAT transgenic line. These lines with the genotype lsp2‐Gal4:attP3B were used as controls (Fig. 1A). As expected, hAAT was detected in hAAT transgenic lines, at a concentration similar to the serum levels of hAAT (Fig. 1 B).

Importantly, we noticed that transgenic lines expressing hAAT lived longer than that of the control lines. Subsequent lifespan testing proved that both of hAAT‐expressing fruit fly lines lived significantly longer than two control lines with matched genetic background (Fig. 1C). The median age for two hAAT lines were 57 and 51 compared to 42 and 39 for two control lines. To better analyze the data, we pooled AAT lines and control lines, respectively. The percentages of survival at day 42 were 85.7 ± 2.3% for pooled hAAT lines and 25.0 ± 4.5% for combined control lines (Fig. 1D). We performed additional survival experiments to confirm this effect. The lifespan of both female and male was significantly extended in hAAT transgenic flies compared to control flies (Fig. S1A,B). Our results from multiple studies with three control lines and two hAAT lines have shown that hAAT has anti‐aging effect in *Drosophila*. In addition, we also compared AAT‐1 to UAS‐hAAT:+ control and +:Lsp2‐Gal4 control. The lifespan of AAT‐1 was significantly longer than +:Lsp2‐Gal4 control. However, UAS‐hAAT:+ control also survived longer compared to +:Lsp2‐Gal4 (Fig. S1C,D). This experiment was repeated, and similar result was observed (Fig. S1E,F). The lifespan extension effect observed in UAS‐hAAT:+ lines could due to hAAT leakage (Fig. S1G).

### Inhibition of inflammation‐associated pathways in hAAT transgenic *Drosophila*

**Figure 2:**
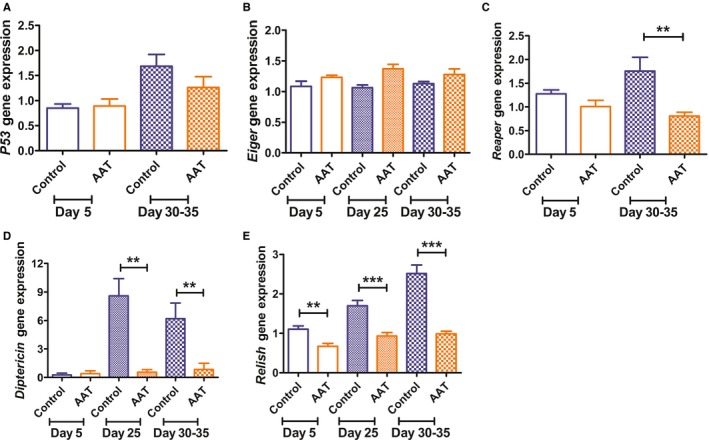
Effect of hAAT on gene expression. Relative levels of mRNA were detected by quantitative RT–PCR for the following genes: (A) *p53*, (B) *Eiger*, (C) *Reaper*, (D) *Diptericin*, (E) *Relish*. *Diptericin* expression was analyzed by one‐tailed Mann–Whitney test. All other data were analyzed by one‐tailed unpaired *t*‐test. *Y*‐axis represented the relative gene expression level. **P ˂ 0.01, ***P ˂ 0.001, *n* = 3, each group contain 6–12 flies/vial.

To probe for the possible mechanisms responsible for the anti‐aging effect of hAAT in transgenic *Drosophila*, we set out to test the impact of hAAT on the expression of a panel of aging‐related genes. P53 has been linked with aging in mammalian system. Like its mammalian counterpart, *Drosophila* P53 (dP53) plays a pivotal role in mediating DNA damage‐induced apoptosis and has been found to be involved in regulating aging (Waskar *et al*., 2009). Our testing indicated that there was an increase in *dP53* expression in older animals. However, this age‐related increase in *dP53* expression was not significantly suppressed by hAAT (Fig. 2A). There was also no significant change in the expression of *eiger*, the TNF orthologue (Fig. 2B). The expression of the stress‐responsive pro‐apoptotic gene, *reaper*, was significantly suppressed by hAAT in older animals (Fig. 2C).

When we tested the effect of hAAT on aging‐related inflammatory genes expressions, we found that both *Relish* (an *NF‐ĸB* orthologue in *Drosophila*) and *Diptericin* (an antimicrobial peptide regulated mainly by Relish) were suppressed by hAAT in aging animals. In the control lines, the level of *Diptericin* mRNA increased about six‐ to eightfold in aged animals (day 25, days 30–35). This increase was largely blocked in hAAT‐expressing animals (Fig. 2D). As shown in Fig. 2E, the expression of *Relish* also increased significantly with aging, which was significantly suppressed by hAAT. While the inhibitory effect of hAAT on *Relish* was most pronounced in older animals (30–35 days old; Fig. 2E), the difference was even significant in young animals, indicating the effect of hAAT on *Relish* gene expression is not limited to aged animals.

### Geneswitch drivers reaffirm that systemic hAAT correlates with extended lifespan of *Drosophila*

**Figure 3:**
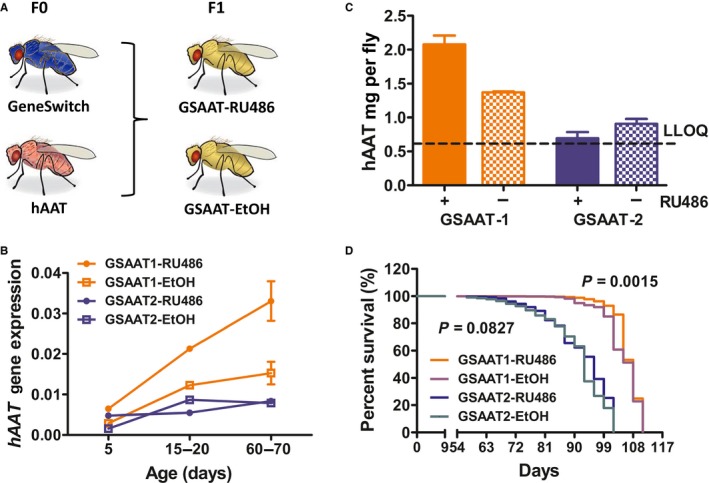
Conditioned expression of hAAT improved lifespan in Geneswitch‐Gal4‐UAS‐hAAT transgenic *Drosophila*. (A) Geneswitch‐Gal4 homozygous male was crossed with UAS‐hAAT homozygous virgins. F1 male flies were collected and raised on food supplemented with either 160 μg mL^−1^ of RU486 or same volume of 100% EtOH. (B) hAAT gene expression measured by quantitative RT–PCR at age of days 5, 15–20, and 60–70. Orange and blue represent GSAAT1 and GSAAT2 lines, respectively. Circle and square represent groups fed on food supplemented with RU486 or EtOH, respectively. For each detection, 3–6 flies were used. For day 5, 1–2 detections, for days 15–20, one detection, and for day 60, six detections. (C) hAAT protein level in 6‐day‐old male flies as measured by ELISA. Each bar represents the average data from two detections (8–25 flies per detection). Color code is the same as in B. The dash line indicates lower limit of quantification (LLOQ). (D) Survival curve of Geneswitch‐Gal4‐UAS‐hAAT transgenic male flies (*n* = 78–142). Percentage of survival was analyzed by the log‐rank (Mantel–Cox) test, and the respective *P* values were indicated next to the survival curves.

A potential caveat of our analysis is that the genetic background of the AAT and control lines might be different even though they were similarly recombined. To solve this problem, we used the Geneswitch system whereby the DNA binding domain of Gal4 is fused with the ligand binding domain of progesterone receptor and thus making it responsive to RU‐486. Two Geneswitch‐Gal4 lines (P{w[+mW.hs]=Switch2}GSG6710 and P{w[+mW.hs]=Switch2}GSG10751) have been reported to drive fat body‐specific expression and were tested together in parallel. Both lines were generated by the same study but have their P element inserted on different chromosomal location (Nicholson *et al*., 2008). These two lines were crossed with the same UAS‐hAAT line, and males of the F1 progeny were used for lifespan analysis. For simplicity, we will refer F1 males from these two crosses as GSAAT‐1 and GSAAT‐2, respectively (Fig. 3A).

We first evaluated transgene (hAAT) expression with both Q‐PCR (Fig. 3B) and ELISA (Fig. 3C). There was significant difference in terms of levels of hAAT expression between GSAAT‐1 and GSAAT‐2 flies, possibly reflects the different chromosomal environment. GSAAT‐2 had very little leaky expression, but the level of hAAT following induction was low as well. Leaky hAAT expression in GSAAT‐1 was apparent, but RU‐486 treatment did increase the level of expression significantly. RU486 treatment significantly increased the lifespan of GSAAT‐1 (RU486 vs. EtOH, *P* = 0.0015). It is remarkable that more than 50% of GSAAT‐1 flies treated with RU486 survived more than 100 days. The same RU486 treatment failed to significantly increase the lifespan of GSAAT‐2 flies (RU486 vs. EtOH, *P* = 0.0827), but the trend was consistent with lifespan extension (Fig. 3D).

Interestingly, the lifespan of GSAAT‐1 was significantly longer than that of GSAAT‐2. Median age for GSAAT‐1 and GSAAT‐2 treated with RU486 was 108 and 96, respectively. The maximum age for these two lines was 111 and 102, respectively. As the two Geneswitch driver lines were generated from the same genetic background, it seems that the difference in lifespan may be very likely due to the different levels of hAAT expressed in these animals. Overall, our analysis using the Geneswtich system reaffirmed that increased systemic hAAT levels correlates with extended lifespan within a dosage ranged of 2.5 ng per fly.

### Systemic hAAT specifically suppresses NF‐kB‐regulated immune‐responsive genes

**Figure 4:**
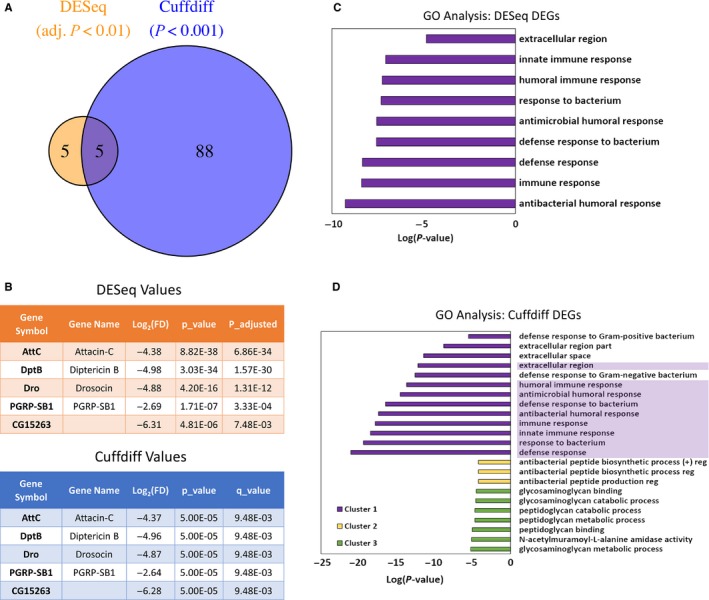
Systemic hAAT specifically suppressed immune‐responsive genes. The global gene expression profiles in hAAT transgenic and control lines at days 30–35 of male flies were analyzed by RNA‐seq. (A) Comparison of differentially expressed genes (DEGs) identified by DESeq (adj *P* < 0.01, left panel) and Cuffdiff (*P* < 0.001, right panel). Five DEGs were identified by both methods. (B) List of the five overlapping genes and respective *P* values calculated by DESeq (upper panel) and Cuffdiff analyses (lower panel). (C) Gene ontology (GO) clustering analysis with DESeq identified DEGs. (D) GO clustering analysis with Cuffdiff identified DEGs. Highlighted terms in figure (D) are also present in figure (C).

To gain a comprehensive picture of genes affected by systemic hAAT, we performed RNA‐seq expression profiling using 30–35 days Gal4‐UAS transgenic flies. Differentially expressed genes (DEGs) were identified by both Cuffdiff and DESeq analysis. Cuffdiff identified 60 down‐regulated and 33 up‐regulated genes (*P*‐value < 0.001) (Fig. 4A). With the more stringent methodology of DESeq, 10 DEGs were identified (adjusted *P* < 0.01), all of which were down‐regulated. Five genes were recognized by both methods (Fig. 4B). Interestingly, the six top‐ranked genes identified by DESeq analysis and four of the five genes identified by both methods encode antimicrobial effector genes.

We next performed GO analysis with the significant DEGs. For genes identified by DESeq analysis, we found nine terms significantly enriched, all of which were associated with immunity and inflammation, including ‘antibacterial humoral response’ (GO:0019730), ‘immune response’ (GO:0006955), and ‘innate immune response’ (GO:0045087) (Fig. 4C). For DEGs identified by Cuffdiff, three clusters of 23 GO terms were significantly enriched. The top‐ranked 10 terms were related to inflammatory response (Fig. 4D), including all nine terms found to be significantly enriched for DEGs identified by DESeq. These results clearly demonstrated that hAAT specifically inhibited the expression of immune‐responsive genes in aged *Drosophila*.

### Treatment of hAAT inhibited SASP from senescent human fibroblasts

**Figure 5:**
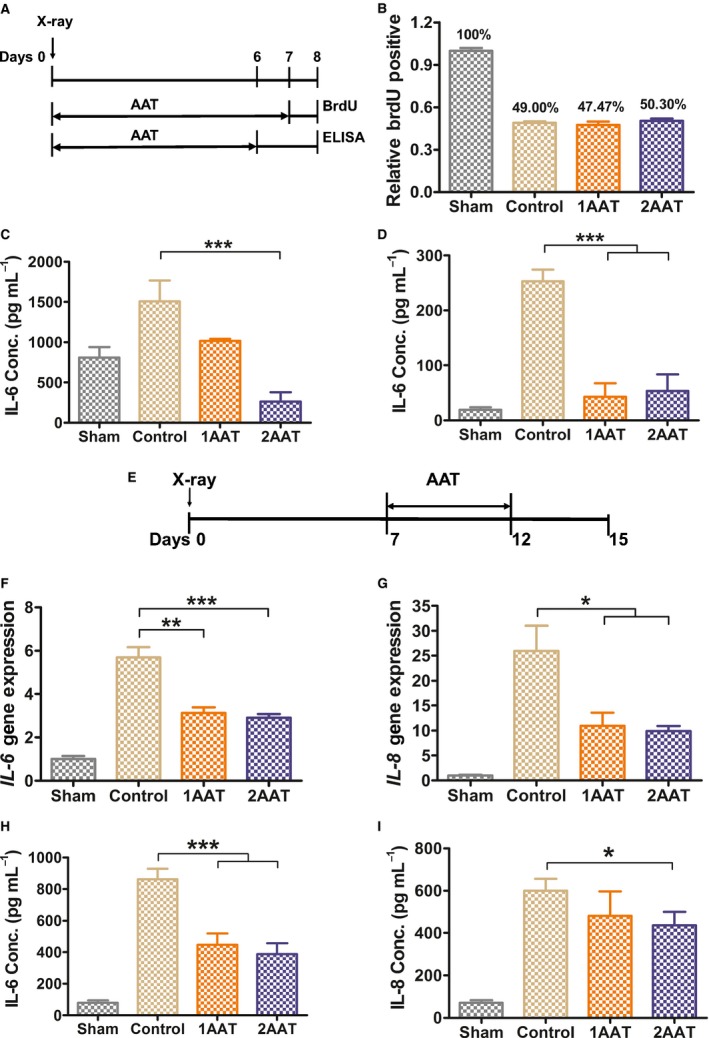
Treatment of hAAT suppresses senescence‐associated secretory phenotype (SASP). (A) Experimental design to test the effect of AAT on senescence and SASP. Immediately after irradiation, HCA2 cells were cultured with hAAT at 1 mg mL^−1^ (1AAT) or 2 mg mL^−1^ (2AAT) or PBS (control) for 6–7 days. BrdU was added at day 7. Flow cytometry was performed to measure the BrdU incorporation at day 8. For the detection of IL‐6 secretion, medium was collected at day 6; cells were thoroughly washed and cultured in fresh serum‐free medium for 2 days. Serum‐free medium was collected at day 8 for IL‐6 detection. (B) Percentage of proliferating (BrdU positive) cells (*N* = 3). (C) IL‐6 concentration in medium at day 6 (*N* = 4). (D) IL‐6 concentration in medium at day 8 (*N* = 4). (E) Experimental design to test the effect of hAAT on SASP after cell senescence. Seven days after irradiation, hAAT or PBS was added. After 5‐day treatment, the cells were washed and fresh medium without treatment was added. Medium or mRNA was harvested at day 15. (F) Relative gene expression of *IL‐6* (*N* = 3). (G) Relative gene expression of *IL‐8* (*N* = 3). (H) IL‐6 concentration in culture media (*N* = 6). (I) IL‐8 concentration in the culture media (*N* = 6). IL 6 and IL‐8 concentrations in the culture medium were detected by ELISA. Gene expression (mRNA) levels of *IL‐6* and *IL‐8* were detected by real‐time RT–PCR. Data of IL‐8 concentration were analyzed by two‐tailed *t*‐test. All the other data were analyzed by one‐way ANOVA followed by Bonferroni comparison. *P ˂ 0.05, **P ˂ 0.01, ***P ˂ 0.001.

As inflammaging is closely related to SASP (Lopez‐Otin *et al*., 2013), we tested the anti‐SASP effect of hAAT in human cells. We irradiated HCA2 cells (human foreskin fibroblasts) to induce cell senescence (Rodier *et al*., 2009; Freund *et al*., 2011; Kang *et al*., 2015; Laberge *et al*., 2015). To test the effect of hAAT on senescence formation, HCA2 cells were treated with either hAAT (1 mg mL^−1^ or 2 mg mL^−1^) or 1× PBS (Fig. 5A) immediately after X‐irradiation (10 Gy, 2.56 Gy min^−1^). BrdU labeling showed that hAAT treatment did not interfere with irradiation‐induced cell senescence (Fig. 5B). However, the treatment significantly inhibited irradiation‐induced IL‐6 secretion (Fig. 5C). This suppression effect remained significant even after the withdrawal of hAAT (Fig. 5D).

We next tested whether hAAT can suppress SASP after cells had entered senescence. Following irradiation, HCA2 cells were cultured for 7 days to allow completion of senescence (Coppe *et al*., 2008). We then treated the cells with or without hAAT for 5 days (Fig. 5E). Main SASP factors, IL‐6 and IL‐8, were suppressed in both transcription and protein level (Fig. S2B). To avoid the possibility that hAAT directly interferes with SASP factors, we washed the cells thoroughly and cultured using fresh serum‐free medium without hAAT for 3 days (Fig. 5E). The gene expression and secretion of major SASP factors, IL‐6 (Fig. 5F,H) and IL‐8 (Fig. 5G,I), were significantly decreased in hAAT‐treated groups than those in control groups. This inhibitory effect of hAAT remained detectable 7 days after hAAT withdrawal (Fig. S2A,C). These observations clearly demonstrated that hAAT can inhibit the transcription and secretion of SASP in senescent cells.

**Figure 6:**
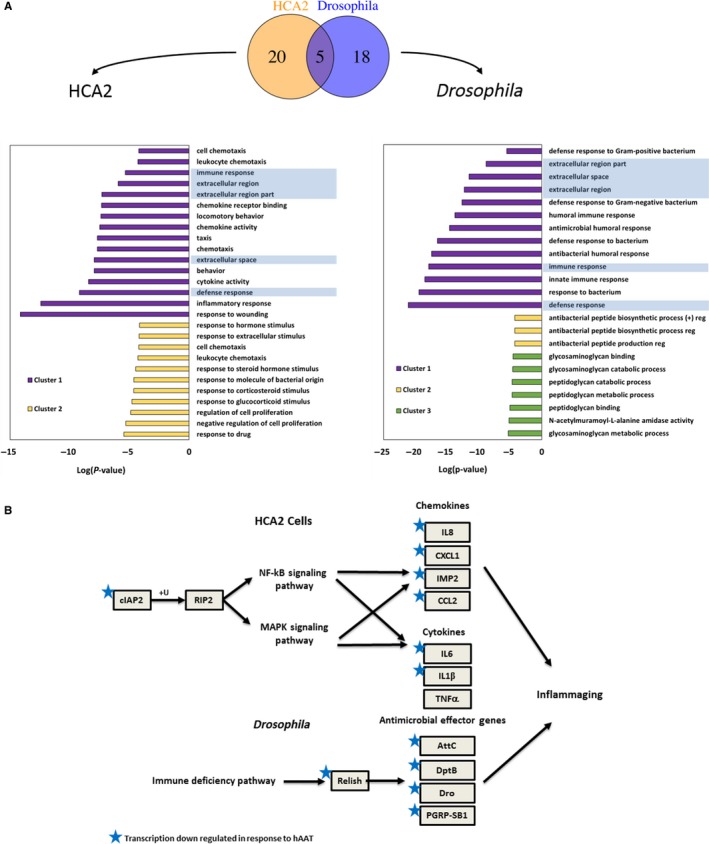
HAAT inhibits inflammaging pathways in both *Drosophila* and HCA2 cells. (A) Comparison of enriched gene ontology (GO) terms through GO clustering analysis in both *Drosophila* and HCA2 cells. Highlighted terms are identical in both *Drosophila* and HCA2. GO terms that had log (*P*) values < −4 were plotted. Only down‐regulated genes led to significantly enriched GO terms (B) Significantly down‐regulated genes and the relevant pathways in both *Drosophila* and HCA2 cells.

To understand the overall impact of hAAT on senescent cells, we performed RNA‐seq analysis in hAAT‐treated senescent cells at day 15 (Fig. 6A). Results from this expression profiling experiment showed that nine genes were significantly up‐regulated and 33 genes were significantly (*P* < 0.001) down‐regulated in hAAT‐treated group compared to control group. The down‐regulated genes include eight of SASP genes (*IL‐6*,* IL‐8*,* CCL2*,* CCL7*,* IL1Beta*,* CXCL1*,* CXCL2,* and *CXCL6*). We also performed GO analysis and identified two clusters of 25 unique terms that were significantly enriched among the DEGs [Lg(*P*‐value) < −4] (Fig. 6A). DEGs used for clustering included *P* < 0.001 and at least one RPKM greater than or equal to 5. The mostly significant GO terms are ‘response to wounding’ (GO:0009611, log *P*‐value = −14.14) and ‘inflammatory response’ (GO:0006954, log(*P*‐value) = −12.42). In addition, many other significantly enriched terms were also related to inflammation, such as ‘cytokine activity’ (GO:0005125, Log *P*‐value = −8.41) and ‘chemokine activities’ (GO:0008009, log *P*‐value = −7.47). Interestingly, when significantly enriched GO terms for genes suppressed by hAAT treatment in HCA2 cells were compared with those identified for genes suppressed by systemic hAAT in *Drosophila*, five terms were identified in both models and two of them were directly associated with inflammation. Together, these data clearly demonstrated hAAT treatment inhibited SASP gene expression and common inflammaging‐related pathways in both human cells and *Drosophila* (Fig. 6B).

## Discussion

Increasing evidence has shown that inflammaging is destructive and may contribute to the pathogenesis of many aging‐associated diseases (Franceschi & Campisi, 2014). However, it is challenging to control the progress of inflammaging. In several animal models of inflammation related diseases, hAAT has been shown to have remarkable effect on delaying or blocking the onset of diseases or alleviating the symptoms (Ma *et al*., 2010; Cao *et al*., 2011; Grimstein *et al*., 2011; Moldthan *et al*., 2014; Akbar *et al*., 2016; Elshikha *et al*., 2016). In fact, some of them are age‐associated diseases, such as arthritis and osteoporosis. However, the direct effect of hAAT on inflammaging has never been tested. Our study, for the first time, showed that hAAT has anti‐inflammaging effect in both *Drosophila* and human cell models. These results indicate that hAAT is either directly involved in or interact with a highly conserved mechanism that plays a central role in controlling inflammaging.

We used two *Drosophila* transgenic models to demonstrate the lifespan extension effect of hAAT. Our data using Gal4‐UAS‐hAAT transgenic system showed significant lifespan extension effect of hAAT in both male and female. Utilization of the Geneswitch‐Gal4‐UAS system excluded genetic background variation and showed RU486 treatment in GSAAT‐1 line‐induced hAAT expression and significant improvement in lifespan. We note that the difference we observed may not reflect the whole strength of hAAT as there was significant leakage of *hAAT* expression in flies only supplemented with ethanol. A tighter Geneswitch driver is needed to reveal the full capacity of hAAT in inhibiting inflammaging. The significantly longer lifespan of GSAAT‐1 compared to GSAAT‐2 is also consistent with the positive effect of hAAT on lifespan extension. Together, our results showed that overexpression of hAAT in *Drosophila* can significantly extend the lifespan.

Given what was known about the function of hAAT in mammalian models, the anti‐aging effect of hAAT could result from additive or synergetic effect of different aspects of hAAT functions, including immunoregulatory, anti‐inflammatory, and cytoprotective functions observed in various animal models. However, our transcriptomic analyses indicated that immune‐responsive genes, especially those regulated by NF‐ĸB (Relish), are specifically suppressed by hAAT. Aging‐related increase in antimicrobial genes has been observed in *Drosophila* by previous studies (Highfill *et al*., 2016). Although the cause of this inflammaging phenotype remained unclear, our finding suggests that increased expression of *Relish* could be an important underlying cause of the inflammaging phenotype associated with aged *Drosophila*. The importance of Relish in controlling inflammaging associated with neurodegenerative diseases has been documented before (Kounatidis *et al*., 2017). It has also been shown that inhibiting the IMD/Relish pathway in old intestine enterocytes prevented dysbiosis, reduced dysplasia, and extended the lifespan (Guo *et al*., 2014). An extensive QTL mapping study found that the chromosomal interval containing *Relish* has the strongest impact on lifespan of *Drosophila* (Highfill *et al*., 2016). The same study also found that the expression level of *Relish* was significantly higher in old (median lifespan) animals compared to young (1–3 days) animals. A recent study found IMD/NF‐ĸB/Relish immune signaling in brain played a critical role in determining lifespan and age‐related neurodegeneration. The same study also indicated that elevation of brain IMD/NF‐ĸB signaling by targeting its intracellular negative regulator led to increased brain AMP gene expression, shorter lifespan, early neurodegeneration, and locomotor decline. In contrast, lowering NF‐ĸB led to extended lifespan as well as enhanced activity (Kounatidis *et al*., 2017). In our hAAT transgenic strain, the expression level of *Relish* is suppressed even in young (day 5) animals (Fig. 2E), while the suppression of Relish targeted genes such as *Diptericin* was only significant in old animals. Kounatidis *et al*. observed shortened lifespan and locomotor decline in flies with brain overexpression of AMPs, including *diptericin*,* drosocin*, and *attacinC* (Kounatidis *et al*., 2017). The expressions of all of these AMPs gene were found to be inhibited by hAAT in our study by either quantitative RT–PCR or RNA‐seq. These evidences suggest that hAAT limited age‐dependent increase in innate immune response genes through inhibiting of *Relish*. This hypothesis, and the underlying mechanism, remains to be studied.

The anti‐inflammaging effect of systemic hAAT observed in *Drosophila* was corroborated by our analysis of SASP in a well‐established mammalian cellular senescence model. Senescent cells accumulate with age in nearly all tissues/organs and produce SASP factors including soluble signaling factors (interleukins, chemokines, and growth factors), proteases, and secreted insoluble proteins/extracellular matrix (ECM) components (Franceschi & Campisi, 2014). Among all the SASPs, pro‐inflammatory cytokines and chemokines are important components that could contribute to inflammaging and aging‐associated diseases (Coppe *et al*., 2010). For instance, senescence of pulmonary artery smooth muscle cells may induce pulmonary hypertension by enhancing proliferation and migration of surrounding smooth muscle cells due to IL‐6 and IL‐8 secretion (Noureddine *et al*., 2011). A recent study using longitudinal cohort data found higher levels of IL1Beta and IL‐6 were correlated to age‐related illnesses and shorter lifespan (Furman *et al*., 2017). Another study showed inhibition of serum IL‐1β alleviated gout‐related symptoms (aging‐related inflammatory disease) in monosodium urate (MSU) crystals‐induced murine model (Goldberg *et al*., 2017). Therefore, inhibition of SASP will be critical for limiting inflammaging. In this study, we employed the X‐ray‐induced senescence model to characterize hAAT functions. This model does not rely on stimulatory molecules, which may interact with hAAT and complicate the interpretation. It has been previously demonstrated that in this model, senescent cells produce high levels pro‐inflammatory cytokines through the activation of the NF‐ĸB pathway (Rodier *et al*., 2009; Freund *et al*., 2011; Kang *et al*., 2015; Laberge *et al*., 2015). We showed that hAAT treatment significantly decreased the gene expression and secretion of major SASP factors, including IL‐6 and IL‐8. RNA‐seq data also indicated gene expression *IL1Beta*, coding SASP factor IL‐1β, was also inhibited by hAAT. More importantly, we showed that AAT treatment is effective in limiting SASP even after the cells have already entered senescence.

Although there is considerable evolutionary distance between fruit fly and human, we found that hAAT specifically inhibited immune‐responsive genes in both system (Fig. 6). For *Drosophila*, all of the significantly suppressed genes are targets of NF‐ĸB (Relish). The fact that *Relish* expression is suppressed even in young animals expressing hAAT suggests that hAAT may regulate *Relish* expression directly in *Drosophila*. In contrast to the abundance of work on post‐translational modification and activation of NF‐ĸB, very little is known about the transcriptional control of NF‐ĸB expression and how elevated expression of NF‐ĸB may contribute to inflammaging in *Drosophila*. NF‐ĸB was found activated in multiple cell types in both wild‐type mice and the XFE progeroid syndrome mice (characterized by accelerated aging induced by accumulated stochastic endogenous DNA damage). Inhibition of NF‐ĸB by either depletion of one allele of NF‐ĸB‐p65 subunit or administration of IKK inhibitor resulted in delayed onset of aging‐related phenotypes in progeroid syndrome mice. Also, decreasing NF‐ĸB‐p65 activity altered cellular senescence *in vitro* and reduced senescent cell *in vivo* (Tilstra *et al*., 2012).

In summary, we have shown that hAAT has potent anti‐inflammaging function in transgenic *Drosophila* as well as DNA damage‐induced senescent cells. Although the detailed mechanism awaits further investigation, data from both systems point to the inhibitory effect of hAAT on aging‐related NF‐ĸB gene expression and activation. As hAAT is a FDA‐approved drug with a confirmed safety profile, the anti‐inflammaging function may make hAAT a promising candidate to combat aging and aging‐related diseases.

## Experimental procedures

### *Drosophila* lines

The Gal4 driver lsp2‐Gal4 was obtained from the Bloomington stock center (Stock # 6357). To generate transgenic lines carrying UAS‐hAAT, hAAT cDNA was cloned into the pUASTattB vector, followed by injection into the VK00033 strain with the attP‐3B docking site (Bloomington stock # 24871, injection performed by Rainbow Transgenic Flies, Inc., Camarillo, CA, USA). Two fly strains resulting from independent integration events were used for recombination with the lsp2‐Gal4 to generate lsp2‐Gal4:UAS‐hAAT. The docking strain was recombined with the Gal4 strain to generate fly with the genotype lsp2‐Gal4:attP. Two independent recombination lines were used as controls for this study. Two Geneswitch strains were purchased from Bloomington (stock # 59926 and 59939). Fly stocks were raised on Nutri‐Fly Bloomington Formulation media (Genesee Scientific Cat # 66‐112, San Diego, CA, USA) and kept at 25 °C under a 12‐h/12‐h light/dark cycle.

### Lifespan experiment

For data presented in Fig. 1, The male and female progenies of the indicated genotype were separated within 12 h of eclosion. Flies were kept at a density of 8–12 flies per vial at 25 °C, 12‐h/12‐h light/dark cycle, and 40–60% humidity. Flies were transferred to fresh food medium every 9 days for Gal4‐UAS study, every 3 days for GSAAT study, and every 3 days for AAT‐1 (Gal4‐UAS‐hAAT)/UAS‐hAAT:+/+:Lsp2‐Gal4 study.

Geneswitch Gal4 lines, P{w[+mW.hs]=Switch2}GSG6710 (Stock# 59926) and P{w[+mW.hs]=Switch2}GSG10751 (Stock # 59939) were obtained from the Bloomington stock center. Both lines were crossed with the same UAS‐AAT line. F1 males, designated as GSAAT‐1 and GSAAT‐2, respectively, were selected at 1st day following eclosion and randomly separated into groups of 10–12. Flies were cultured with Nutri‐Fly media supplemented with either 160 μg mL^−1^ of RU486 (Cayman Chemical, Ann Arbor, MI, USA, Item #: 10006317) dissolved in ethanol or same volume of ethanol.

### Cells and cell culture

HCA2 cells were a gift from Dr. Judith Campisi. Fibroblast were cultured in Minimum Essential Medium Eagle (MEM) (Sigma, St. Louis, MO, USA, Cat #: M4526) with 10% fetal bovine serum (FBS) (Atlanta Biologicals, Lawrenceville, GA, USA, Cat #: S11150H), 1× penicillin/streptomycin, and 2 mm l‐glutamine.

### Senescence induction and assessment

Cells were subjected to 10 Gy (2.56 Gy min^−1^) of X‐irradiation using X‐RAD 320 (Precision X‐ray Inc., North Branford, CT, USA). Control cells were sham operated in parallel with the treated cells. The irradiated cells were treated with either 1XPBS or hAAT (high dose: 2 mg mL^−1^; low dose: 1 mg mL^−1^) for 7 days. BrdU incorporation was assessed using FITC BrdU Flow Kit (BD Biosciences, San Jose, CA, USA, Item #: 559619) according to the protocol provided by the manufacturer. The experiments were performed in triplicates.

### RNA extraction and Q‐PCR

Fly and fibroblast RNA were extracted with Quick‐RNA MiniPrep (Genesee Scientific, Cat #: 11‐328) according to the protocol provided by the manufacturer. cDNA was prepared by reverse transcription of total RNA with a High‐Capacity cDNA Archive Kit (Applied Biosystems, Foster City, CA, USA, Ref #: 4368814). Q‐PCR was performed with an ABI 7500 Fast thermocycler (Applied Biosystems, Ref #: 4368813) following protocols provided by the manufacturer. Triplicates were processed for each gene/sample combination. The primers were listed as following. Relish forward: 5′‐GGCATCATACACACCGCCAAGAAG‐3′, Relish reverse: 5′‐GTAGCTGTTTGTGGGACAACTCGC‐3′, Diptericin forward: 5′‐ATTGGACTGAATGGAGGATATGG‐3′, Diptericin reverse: 5′‐CGGAAATCTGTAGGTGTAGGT‐3′, Eiger forward: 5′‐CTGCCGAGACCCTCAAGC‐3′, Eiger reverse: 5′‐AGATCGTTAGTGCGAGAATG‐3′, D‐GAPDH forward: 5′‐AAGGGAATCCTGGGCTACAC‐3′, D‐GAPDH reverse: 5′‐CGGTTGGAGTAACCGAACTC‐3′, IL‐6 forward: 5′‐TACCCCCAGGAGAAGATTCC‐3′, IL‐6 reverse: 5′‐TTTTCTGCCAGTGCCTCTTT‐3′, IL‐8 forward: 5′‐GTGCAGTTTTGCCAAGGAGT‐3′, IL‐8 reverse: 5′‐CTCTGCACCCAGTTTTCCTT‐3′, RPL13A forward: 5′‐GTACGCTGTGAAGGCATCAA‐3′, RPL13A reverse: 5′‐CGCTTTTTCTTGTCGTAGGG‐3′, hAAT forward: 5′‐CTGAATTTCAACCTCACGGAGAT‐3′, hAAT reverse: 5′‐GGTTGAGGGTACGGAGGAGTT‐3′.

### RNA sequencing

The RNA‐seq libraries for both *Drosophila* and HCA2 samples were prepared using TruSeq Stranded mRNA Library Prep Kit (Illumina, San Diego, CA, Cat #: RS‐122‐2101), according to the protocol provided by the manufacturer. The mixed libraries were sequenced with Illumina NextSeq 500. About 21 million reads were obtained for *Drosophila* samples, and about 60 million reads were obtained for every HCA2 sample. DEGs for *Drosophila* samples were identified using the Cuffdiff 2 (Trapnell *et al*., 2013) and DESeq (Anders & Huber, 2010) programs, while Cufflinks analysis was performed for HCA2 cell samples.

### ELISA

To measure the level of hAAT in transgenic flies, 8–30 adults of both gender were homogenized in 0.5 mL or 1 mL of 1× PBS. Supernatant was harvested and processed for hAAT ELISA as previously described (Lu *et al*., 2006). To detect the IL‐6 and IL‐8 secretion in cell culture medium, Human IL‐6 Mini ABTS ELISA Development Kit (Pepro Tech, Rocky Hill, NJ, USA, Cat #: 900‐M16) and Human IL‐8 Mini ABTS ELISA Development Kit (Pepro Tech, Cat #: 900‐M18) were applied according to the protocol provided by the manufacturer.

## Funding

This work was supported in part by NIH grant GM106174 (LZ).

## Conflict of interest

None of the authors has any potential financial conflict of interest related to this manuscript.

## Author contributions

YY, SS, and LZ contributed to all aspects of the study, including the conception and design, data collection and analysis, and manuscript preparation and revision. BD contributed to RNA‐seq data analysis, preparation, and revision of the manuscript. YL, DT, BB, LS, HP, NK, AE, MA, and YL contributed to data collection.

## Supporting information


**Fig. S1** Transgenic expression of hAAT extended lifespan of both female and male.
**Fig. S2** Treatment of hAAT after senescence formation suppressed SASP gene expression and secretion.
