# Supplementary material for: Anti‐inflammaging effects of human alpha‐1 antitrypsin
Source: Aging Cell. 2017 Oct 17;17(1):e12694. doi: 10.1111/acel.12694 (PMC5770780; doi:10.1111/acel.12694)
Supplement: Supplementary file 1 — Fig. S1 Transgenic expression of hAAT extended lifespan of both female and male. Fig. S2 Treatment of hAAT after senescence formation suppressed SASP gene expression and secretion. [file ACEL-17-na-s001.pdf]

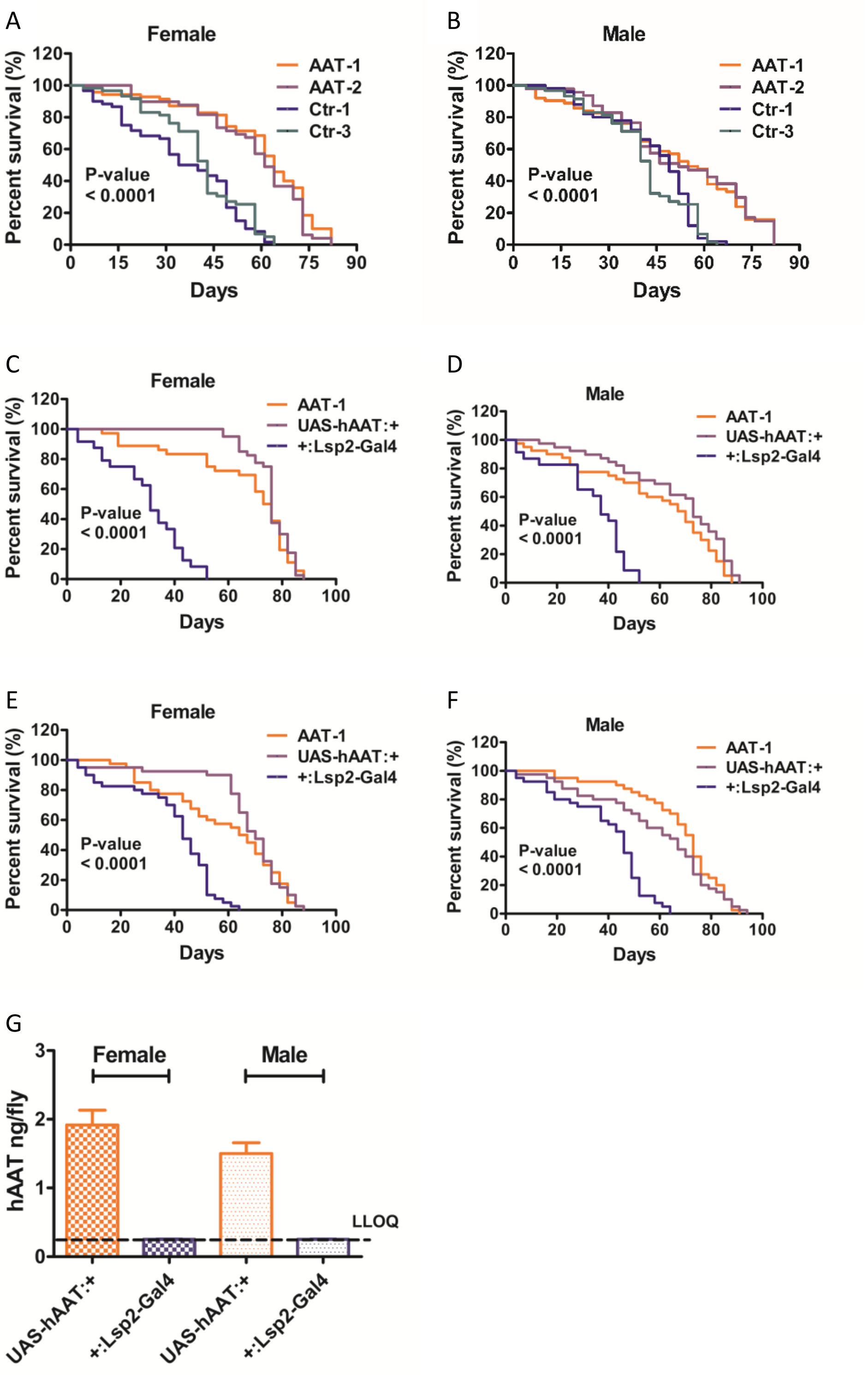

A
Female
Percent survival (%)
Days
AAT-1
AAT-2
Ctr-1
Ctr-3
P-value
< 0.0001
B
Male
C
UAS-hAAT:+
+:Lsp2-Gal4
D
E
F
G
hAAT ng/fly
LLOQ

Fig. S1 Transgenic expression of hAAT extended lifespan of both female and male. A & B. Survival curve of recombined Lsp2Gal4:UAS-hAAT (AAT) and similarly recombined control (Ctr) transgenic lines. n = 49 – 70 for female (A) and n = 47-63 for males (B). **C & D**. Survival curves of flies of the genotype UAS-hAAT:Lsp-Gal4 (AAT-1), UAS-hAAT/+, and +/Lsp2-Gal4. n=24-40 for female (C), n = 23 – 40 for male (D). **E & F**. represent an independently repeated measurement of C & D, respectively. N = 40 for both female (E) and male (D). **G**. hAAT protein level in transgenic flies represented in C-F were measured by ELISA. Each bar represents the average data from 3 detections (25-50 flies per detection). Percentage of survival was analyzed by Log-rank (Mantel-Cox) test.

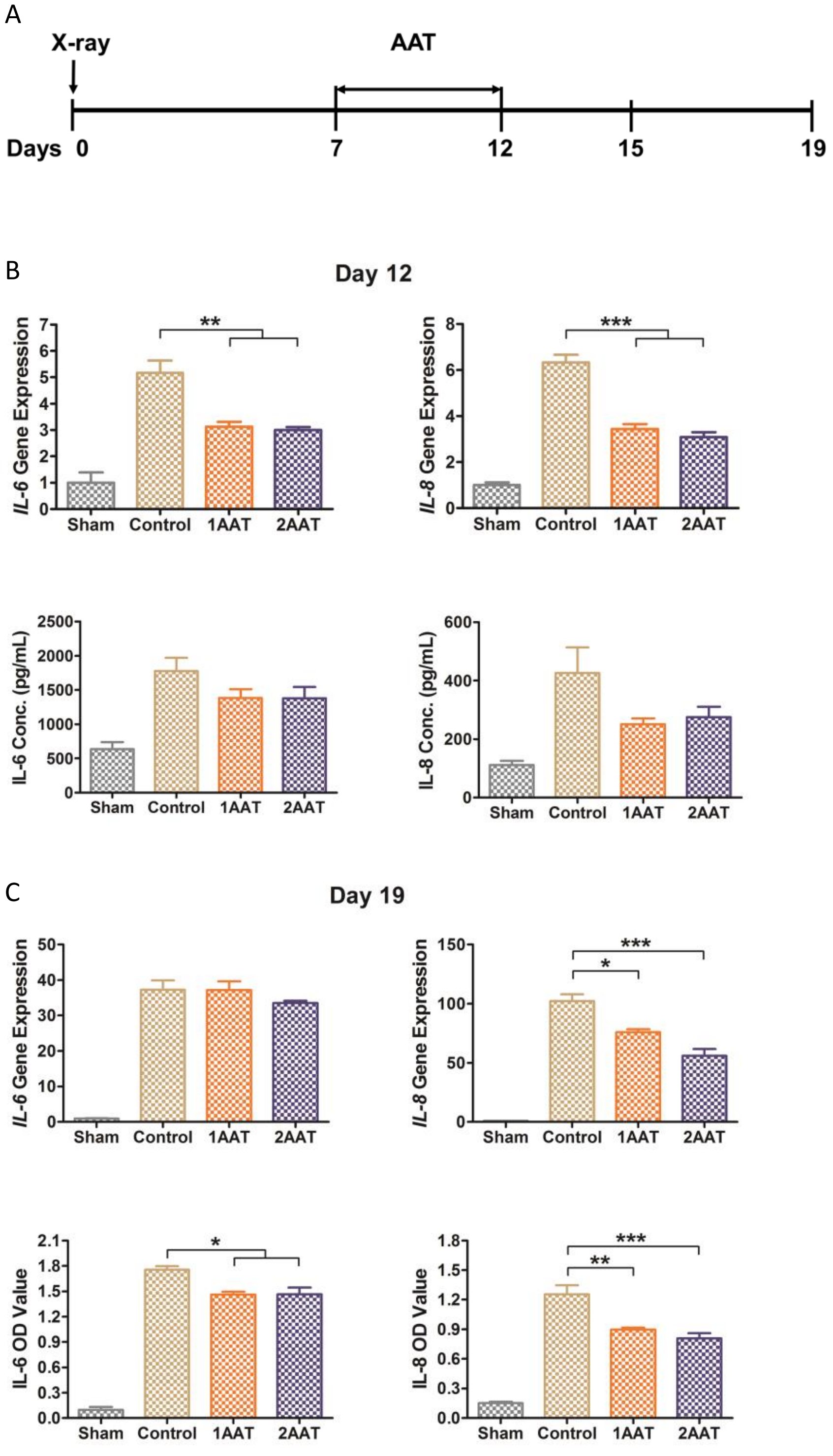
A
X-ray
AAT
Days 0 7 12 15 19
B
Day 12
IL-6 Gene Expression
IL-8 Gene Expression
IL-6 Conc. (pg/mL)
IL-8 Conc. (pg/mL)
Sham Control 1AAT 2AAT
C
Day 19
IL-6 Gene Expression
IL-8 Gene Expression
IL-6 OD Value
IL-8 OD Value
Sham Control 1AAT 2AAT

Fig. S2 Treatment of hAAT after senescence formation suppressed SASP gene expression and secretion. A. Experimental design to test the effect of hAAT on SASP after cell senescence. Seven days after irradiation, hAAT or PBS was added. After 5-day treatment, the cells were washed and fresh medium without treatment was added. Medium or mRNA were harvested at day 12 and day 19. **B**. SASP gene expression and secretion at day 12. **C**. SASP gene expression and secretion at day 19. All the data were analyzed by one-way ANOVA followed by Bonferroni comparison. * $P < 0.05$, ** $P < 0.01$, *** $p < 0.001$.
